# Root Morphology of the Maxillary First and Second Molars in an Iranian Population Using Cone Beam Computed Tomography

**Published:** 2017-05

**Authors:** Zahra Ghoncheh, Behrang Moghaddam Zade, Mohammad Javad Kharazifard

**Affiliations:** 1 Assistant Professor, Department of Oral and Maxillofacial Radiology, School of Dentistry, Tehran University of Medical Sciences, International Campus, Tehran, Iran; 2 Adjunct Professor, Department of Oral and Maxillofacial Radiology, School of Dentistry, Tehran University of Medical Sciences, International Campus, Tehran, Iran; 3 Epidemiologist, Dental Research Center, Dentistry Research Institute, Tehran University of Medical Sciences, Tehran, Iran

**Keywords:** Tooth Root, Cone-Beam Computed Tomography, Maxilla, Molar, Population

## Abstract

**Objectives:**

This study sought to assess the root morphology and root canal anatomy of the maxillary first and second molars in an Iranian population using cone-beam computed tomography (CBCT).

**Materials and Methods:**

Sound fully-developed maxillary first (n=345) and second (n=423) molars were evaluated on 450 CBCT scans ordered for pre-operative assessment for implant placement. The (I) number of roots and their morphology (II) number of canals per root, (III) canal configuration and presence of a second mesiobuccal canal according to the Vertucci’s classification and (IV) unilateral or bilateral occurrence of a second mesiobuccal canal (MB2) were evaluated.

**Results:**

Single roots were found in 1.1% of the first and 11.3% of the second molars. Four separate roots were identified in 0.5% of the first molars; none of the second molars had four separate roots. First and second molars showed a higher prevalence of three separate roots of mesiobuccal, distobuccal and palatal with one canal in each root (54% and 86 %, respectively). The most common anatomical variation in the maxillary first molars was related to the configuration of the MB root; the root canal system of the maxillary second molars showed more anatomical variations.

**Conclusions:**

Mesiobuccal roots of the maxillary molars had more variations in their canal system than the distobuccal or palatal roots. The root canal configuration of the maxillary second molars was more diverse than that of first molars; CBCT enhances mapping of the mesiobuccal root canal system with the potential to improve the quality of root canal treatment.

## INTRODUCTION

Successful endodontic treatment requires adequate knowledge of clinicians about the morphology of the root canals. Lack of knowledge in this regard and missing a root canal are among the most common causes of failure of root canal treatments [1].

Most previous studies on maxillary molars have reported that these teeth usually have three roots and four canals since an extra canal is often found in the mesiobuccal root. Other anatomical variations in the form of an extra C-shaped canal have also been reported in distobuccal and palatal roots. Thus, due to having a more complex anatomy compared to other teeth, maxillary molars have the highest rate of endodontic failure. For this reason, it is imperative for the clinicians to have adequate knowledge about the root anatomy and canal morphology of the teeth [2–5]. Also, racial differences cause variations in the anatomy of the root canal system, which further necessitate assessment of root canal anatomy in different races and ethnic groups [4]. Several studies have assessed the morphology of the roots and root canal anatomy in different populations using different techniques such as sectioning [6], root canal staining and clearing [7], periapical radiography [8] and computed tomography scanning [9]. However, all these techniques have some limitations. For instance, the staining and clearing technique is an in vitro technique and cannot be performed on patients. This technique is only suitable for the extracted teeth; however, collection of extracted teeth is difficult, and bilateral teeth belonging to the same patient are difficult to find. Periapical radiography provides a two-dimensional image of a three-dimensional object, resulting in distortion and superimposition of the images. As the result, some details are missed and the buccal and lingual aspects of the teeth cannot be well visualized [1,8]. The main disadvantage of computed tomography scanning is the relatively high patient radiation dose [10].

In the 1990s, cone beam computed tomography (CBCT) was first used in endodontics and it is now highly demanded by endodontists as an accurate 3D imaging modality. Some previous studies have assessed the root canal morphology of the permanent maxillary and mandibular molars using CBCT and it has been shown that this imaging modality is beneficial for assessing the configuration of root canals and detecting possible differences in canal shapes and morphology [4]. Recently, CBCT was suggested for scrutiny of root canal details three-dimensionally prior to endodontic treatment [1]. Also, CBCT is a reliable imaging modality to find the second mesiobuccal canal (MB2) compared to physical sectioning (which cannot be done in the clinical setting). Moreover, CBCT provides clinicians with valuable information regarding the position of the teeth and configuration of root canals, which can greatly help in non-surgical root canal treatment of teeth [11]. High resolution, significant reduction in patient radiation dose, fast action and low cost are among the main advantages of CBCT [4,11]. Metal artifacts are among the disadvantages of CBCT, which complicate accurate interpretation of CBCT scans. This limitation of CBCT must be taken into account when interpreting the images [4, 11]. This study sought to assess the root morphology and canal anatomy of the maxillary first and second molars in an Iranian population using CBCT. The possibility of unilateral or bilateral occurrence of a second mesiobuccal canal and possibility of its occurrence in two adjacent molars were also assessed.

## MATERIALS AND METHODS

This study was conducted on 450 patients (200 males and 250 females) with a mean age of 40 years (range 30 to 50 years) presenting to a private oral and maxillofacial radiology clinic to obtain CBCT scans as part of preoperative assessment for implant placement. The maxillary first and second molars of patients (345 maxillary first and 423 maxillary second molars) were evaluated on CBCT scans.

The inclusion criteria for CBCT scans were as follows:

1. The entire maxilla from the alveolar crest to the vestibular depth had to be clearly visible on the CBCT scans.
2. Presence of fully erupted maxillary first and second molars
3. Absence of lesions/defects in the maxilla
4. Presence of sound maxillary molars with no root restoration, intracanal post, coronal restoration or prosthetic crown
5. Open apex teeth and those with root resorption or intracanal calcifications were not included.

All CBCT scans were taken using NewTom VG CBCT system (Image Works, Verona, Italy) with standard exposure settings (11×16 cm field of view, 0.3mm voxel size, 110kV, 3.6–5.4s). Milliamperage was automatically (safe-beam) adjusted based on the anatomy of each patient from 1–20 mA. All measurements were made using NNT Viewer software (NNT 2.21; Image Works, Verona, Italy).

This software enables measurements with 0.1mm accuracy. All CBCT scans were viewed by an experienced oral and maxillofacial radiologist and an endodontist in a semi-dark room using NNT Viewer software twice with one-week interval.

The intra- and inter-observer agreements were excellent (kappa coefficient=1). To observe the images, the observers first adjusted the contrast and brightness of the images. Next, at the location of the respective tooth, axial images were reconstructed in such a way that the axial plane was parallel to the inferior border of the mandible.

**Fig. 1:**
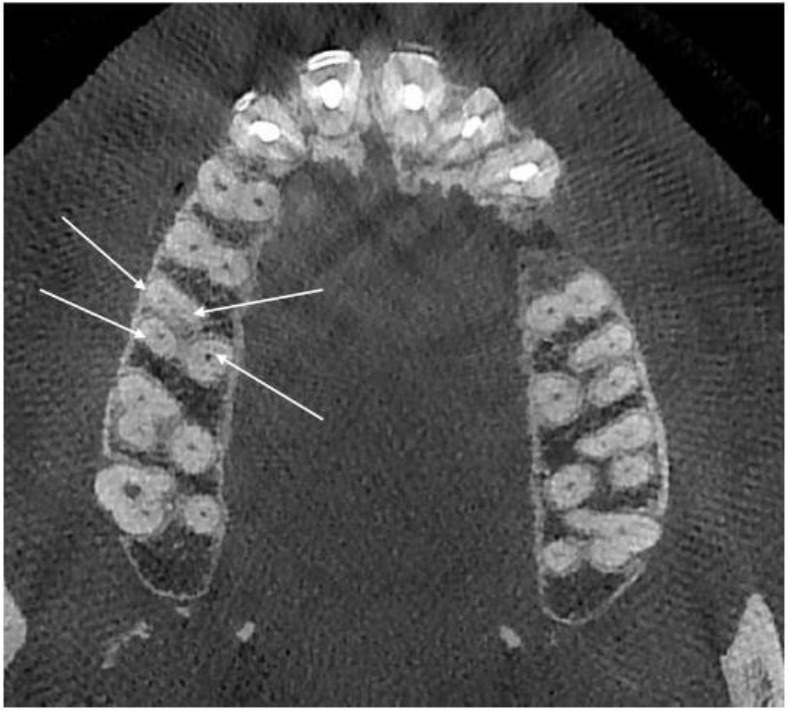
Assessment of the number of roots and their morphology and the number of canals and their configuration on CBCT scan

Then, to assess the anatomy of the root canals of each tooth, the observers scrolled from the coronal towards the apical portion of each tooth. To further scrutinize the root canal anatomy, cross-sectional slices were also studied. All teeth were evaluated in sagittal, axial and coronal planes and the observers evaluated the number of roots and their morphology, the number of canals and their configuration in each root, the possibility of unilateral or bilateral occurrence of MB2 as well as its occurrence in two adjacent molars (Fig. 1).

Number of teeth with one root, two separate roots, three separate roots, two fused roots with one separate root, four separate roots, two-fused and two separate roots, two fused roots and three fused roots was recorded. The configuration of the first and second molar root canals was analyzed using the Vertucci’s classification as follows [7]:

1. One root (types I, II, III, IV, V, VI, VII, VIII)
2. Two roots (buccal and palatal roots) each with types I to VIII
3. Three roots (mesiobuccal, distobuccal and palatal) each with types I to VIII
4. Four roots (a) mesiobuccal, mid buccal, distobuccal and palatal, (b) mesiobuccal, distobuccal, second distobuccal and palatal, (c) mesiobuccal, distobuccal, mesiopalatal and palatal with types I to VIII

## RESULTS

The most common morphology in the maxillary first and second molars was presence of three separate roots with a prevalence of 92.1% (n=318) for the first and 63.3% (n=268) for the second molars.

The anatomical variations of the maxillary second molars were higher than those of the maxillary first molars (Table 1).

**Table 1.** Frequency of anatomical variations of the maxillary first and second molars

	***Maxillary first molars (%)***	***Maxillary second molars (%)***
1S	4 (1.1)	48 (11.3)
2S	2 (0.5)	16 (3.7)
3S	318 (92.1)	268 (63.3)
2F1S	19 (5.5)	78 (18.4)
4S	2 (0.5)	0
2F2S	0	0
2F	0	5 (1.1)
3F	0	8 (1.8)
Total	345	423

1S: One conical root; 2S: Two separate roots; 2F1S: Two fused and one separate root; 4S: Four separate roots; 2F2S: Two fused and two separate roots; 2F: Two fused roots; 3F: Three fused roots

Four separate roots were only seen in two first molars (0.5%) and none of the second molars had four separate roots.

A total of 19 first molars (5.5%) had two fused and one single root. Five second molars (1.1%) had two fused roots and 8 second molars (1.8%) had three fused roots. Four first molars (1.1%) and 48 second molars (11.3%) had one root. The Vertucci’s types for the first and second molars are presented in Tables 2 and 3. The Vertucci’s type I was only observed in four (1.1%) single-rooted first molars and other types were not seen. Of single-rooted second molars (n=48, 11.3%), 43 were Vertucci’s type I, three were type II and two were type III.

**Table 2.** Frequency of the Vertucci’s types for the maxillary first molars

	**Root**	**Type I**	**Type II**	**Type III**	**Type IV**	**Type V**	**Type VI**	**Type VII**	**Type VIII**
Group 1 1 root (n=4)	Single	4	-	-	-	-	-	-	-
Group 2 2 roots (n=2)	B	2	-	-	-	-	-	-	-
	p	2	-	-	-	-	-	-	-
Group 3 3 roots (n=337)	MB	181	50	-	96	10	-	-	-
	DB	328	3	1	4	1	-	-	-
	P	337	-	-	-	-	-	-	-
Group 4 4 roots (n=2)	MB	2	-	-	-	-	-	-	-
	DB	2	-	-	-	-	-	-	-
	MP	2	-	-	-	-	-	-	-
	P	2	-	-	-	-	-	-	-

B: Buccal; DB: Distobuccal; DB2: Additional distobuccal; DP: Distopalatal; MB: Mesiobuccal; Mid B: Mid buccal; MP: Mesiopalatal; P: Palatal

**Table 3.** Frequency of the Vertucci’s types for the maxillary second molars

	**Root**	**Type I**	**Type II**	**Type III**	**Type IV**
Group 1 1 root (n = 48)	Single	43	3	2	-
Group 2 2 roots (n=21)	B	16	4	1	-
	P	21			
Group 3 3 roots (n=345)	MB	305	9	-	40
	DB	354			
	P	354			

B: Buccal; DB: Distobuccal; MB: Mesiobuccal; P: Palatal

Only two (0.5%) first molars had two roots of buccal and palatal, which were both Vertucci’s type I and other types were not seen. There were 21 (4.8%) second molars with two roots of buccal and palatal; the buccal root was type I in 16, type II in four and type III in one tooth.

All 21 teeth had type I palatal root. There were 337 first molars with three roots of mesiobuccal, distobuccal and palatal; the mesiobuccal root was type I in 181, type II in 50, type IV in 96 and type V in 10 teeth. The distobuccal root was type I in 328, type II in four, type III in one, type IV in four and type V in one tooth. The palatal root of all 337 teeth was Vertucci’s type I, and other types were not observed. There were 354 teeth with three roots of mesiobuccal, distobuccal and palatal.

The mesiobuccal root was type I in 305, type II in nine and type IV in 40 teeth. The distobuccal and palatal roots of all 354 teeth were Vertucci’s type I and other types were not seen. There were only two (0.5%) teeth with four roots of distobuccal, mesiobuccal, palatal and mesiopalatal, which were all Vertucci’s type I. None of the second molars had four roots. McNemar’s analysis was performed to assess the possibility of occurrence of an extra mesiobuccal canal (MB2) in two adjacent teeth. As seen in Table 4, the odds of occurrence of a single canal in the mesiobuccal root of a second molar with an adjacent first molar with a single canal mesiobuccal root and the odds of occurrence of two canals in the mesiobuccal root of a second molar with an adjacent first molar with a two-canal mesiobuccal root were significantly higher compared to other situations. The possibility of bilateral occurrence of an extra mesiobuccal canal (MB2) in the first molars and second molars was also assessed using McNemar’s test. The results are presented in Table 5.

**Table 4.** Possibility of occurrence of an extra mesiobuccal canal (MB2) in two adjacent teeth (McNemar’s test)

	Maxillary second molars with MB2	Maxillary second molars without MB2
Maxillary first molars with MB2	32	97
Maxillary first molars without MB2	4	147

(P<0.001)

**Table 5.** Possibility of bilateral occurrence of an extra mesiobuccal canal (MB2) in the first and second molars (McNemar’s test)

	**MB root with additional canal**			**MB root with one canal**	

	**Unilateral**		**Bilateral**	**Bilateral**	**Total**
	**Left**	**Right**			
				
	No. of patients	No. of patients	No. of patients	No. of patients	
Maxillary first molars	16	13	58	45	132
Maxillary second molars	4	-	14	146	164

## DISCUSION

In this stdy, CBCT was used to assess the root morphology and root canal configurations of 345 maxillary first and 423 maxillary second molars in 450 patients.

A total of 318 (92.1%) maxillary first molars had three separate roots, five (5.5%) had two separate and two fused roots, two (0.5%) had four separate roots, two (0.5%) had two separate roots and four (1.1%) had one single root. These findings regarding the prevalence of first molars with three roots are in accordance with the findings of Abed et al, [12] Neelakantan et al, [13] and Al Shalabi et al, [14] using in vitro clearing technique and Thomas et al, [15] using the opaque gel technique in vitro. Rouhani et al. [16] reported a prevalence of 98.4% for three-rooted first molars in an Iranian population, which may be due to the inclusion of teeth with one separate root and two fused roots in the category of three-rooted maxillary first molars; in this case, this rate in our study would reach 97.6%. Kim et al, [1] and Zheng et al. [11] reported the prevalence of three-rooted first molars to be 97.91% and 97%, respectively. They also considered teeth with three separate roots and two-fused roots and one separate root as three-rooted teeth. In a study on a Brazilian population, the prevalence of three-rooted maxillary first molars was reported to be 53%, which may be attributed to racial differences [5]. Some previous studies reported the prevalence of maxillary first molars with three separate roots to be 100% in Burmese [17] and Thai [18] populations, which is probably due to an erroneous assessment method.

With regard to the maxillary second molars, 63.3% of the teeth in our study had three separate roots, 18.4% had two fused and one separate root, 11.3% had one single root, 3.7% had two roots, 1.8% had three fused roots and 1.1% had two fused roots; these values indicate that anatomical variations in the maxillary second molars are significantly more common than in the maxillary first molars. These results revealed that the prevalence of three-rooted second molars in our Iranian population was higher than that in a Brazilian population (45%) [5] and lower than that in Chinese (82%) [2], Korean (75%) [1] and Indian populations (93%) [13].

Rouhani et al, [16] in their study on an Iranian population calculated the prevalence of three-rooted maxillary second molars to be 89.6%, which may be attributed to the fact that we separately assessed the prevalence of maxillary second molars with three fused roots (1.8%) and two fused roots and one separate root (18.4%); if we add up all these values, the prevalence of three-rooted maxillary second molars will reach 83.5%.

Inadequate knowledge about root canal morphology can lead to failure of endodontic treatment. CBCT is a reliable imaging modality to find MB2 compared to other techniques [11]. Several studies have assessed the prevalence of an extra mesiobuccal canal in the maxillary first molars and the existing anatomical variations in this regard [18–20]. The prevalence of the MB2 in the maxillary first molars was 46% in our study; this value was 60% in the study by Kim et al, [1] 68% in the study by Guo et al, [21] 66% in the study by Abed et al, [12] and 65% in the study by Bhuyan et al [22], which are higher than our obtained value. The corresponding values reported by Zhang et al, [2] (52%), Zheng et al, [11] (50%), Neelakantan et al, [13] (49%) and Silva et al, [5] (46%) were close to our obtained value.

The prevalence of the MB2 in the maxillary second molars was 14% in our study, which was lower than the values reported by Silva et al, [5] (34%), Zhang et al, [2] (22%) and Neelakantan et al, [13] (38%). The morphological complexities of the root canals of the maxillary molars were mainly related to the presence of MB2. The anatomy of the mesiobuccal root has been the subject of numerous studies and the prevalence of MB2 in these studies varied from 50% to 80% [14, 17, 23–26]. In our study, MB2 was found in 46% of the maxillary first and 14% of the maxillary second molars.

The most prevalent configuration of MB2 was the Vertucci’s type IV. In our study, MB2 in the maxillary first molars was type IV in 28%, type II in 15% and type V in 3%. Of the maxillary second molars, 11% had type IV and 3% had type II MB2. In the study by Zhang et al, [2] on the maxillary first molars, type IV was the most common type for MB2 (70%) followed by types V (16%) and II (14%). Rouhani et al, [16] in their study on the maxillary first molars reported that Vertucci’s type IV had the highest prevalence for MB2 followed by types II and III. In the study by Kim et al, [1] on the maxillary first molars, types IV and II had a prevalence of 40% and 20%, respectively for MB2. In the study by Neelakantan et al, [13] the prevalence of Vertucci’s type IV for MB2 was 50% in the maxillary first molars and 38.6% in the maxillary second molars.

Guo et al. [21] reported the prevalence of Vertucci’s types IV and II for MB2 to be 42% and 26%, respectively in the maxillary first molars. Abed et al. [12] reported the prevalence of Vertucci’s types IV and II for MB2 to be 35% and 31%, respectively in the maxillary first molars. In the study by Zheng et al, [11] the prevalence of Vertucci’s types IV and II for MB2 was 69% and 14% in the maxillary first molars, respectively. In the study by Bhuyan et al, [22] the prevalence of types IV and II for MB2 was 30% and 28% in the maxillary first molars, respectively and types V and VI were ranked next.

In our study, in 24% of the cases where MB2 was present in the maxillary first molars, the adjacent second molar also had MB2. In cases where the first molar did not have MB2, the adjacent second molar did not have it either in 97% of the cases.

For the maxillary first molars, the prevalence of MB2 in the left quadrant was higher than that in the right quadrant and in 43% of the cases, MB2 canals were present bilaterally; in 34% of the cases, MB2 canals were absent bilaterally.

Micro-computed tomography [23] and clearing and staining techniques [7] reported a higher prevalence for the MB2 canals; however, these modalities can only be used for extracted teeth.

In our study, the prevalence of MB2 canal was higher than the value in studies using periapical radiography, which indicates that CBCT is a more accurate modality for assessment of root canal morphology in patients. Not detecting the MB2 canal is among the major factors contributing to the failure of endodontic treatment.

Moreover, CBCT is a non-invasive modality enhancing endodontic diagnosis since it can greatly help in correct detection of periapical lesions in their first stages of development and can increase the success of treatment compared to conventional radiography.

## CONCLUSION

Within the limitations of this study, it was concluded that more than half of the maxillary first molars in our Iranian population had three roots and four canals. The highest frequency of the fourth canal was found in the mesiobuccal root. Additional canals were often bilateral and CBCT significantly enhanced the detection of fourth canals. These findings can help dentists in easier detection of extra canals in maxillary molars to obtain more favorable results in root canal treatment.
